# Dr. Tice, on the Cure of Intermittents

**Published:** 1804-03-01

**Authors:** C. Tice

**Affiliations:** Blackfriar's Road


### To the Editors of the Medical and Physical Journal.

Gentlemen,

FROM the numerous occasions in which I have experienced the effects of metallic preparations in the treatment of intermittent fevers, is founded a tolerable accurate knowledge of the respective advantages which accrue from their individual use, and a comparative estimation between them and the generally affirmed specific qualities of the cinchona.

The white vitriol I have employed in a number of instances for the cure of agues, with very happy success; but I must confess, from an extensive practice and frequent observation, that it does not possess any specific quality, and that not unfrequently it has failed as a remedy, especially in those cases where the constitution had suffered from repeated attacks of the fever.

From repeated trials, I am induced to prefer the sulphat of copper or blue vitriol to either the solution of arsenic, or the white vitriol. The effects of the latter are neither so certain, nor are they sufficiently lasting in inveterate cases, as those derived from the blue vitriol; and although the blue vitriol and the solutio mineralis arsenici seem to induce equivalent success as to the cure of the disease, or seem to be equally efficacious, yet from the use of the copper copper preparation, seldom any deleterious tendency ensues, whilst some of the worst consequences originate from the administration of the arsenic solution.

In those constitutions which have been much habituated to agues, I have experienced the entire failure in the cure of the fever, from all three of the aforementioned preparations, and the cure only to be effected by the exhibition of the cinchona. From the great number of men labouring under agues, (when the Guards were in Ireland and in the western part of the country, there were seldom fewer than two hundred affected with intermittents and dysenteries for many months) and the continued prevalence of the causes which induced the fever, it was found practicable and necessary to give every remedy a fair and comparative trial; although the Peruvian bark seldom failed to afford a certain remedy, yet, both on account of the difficulty of procuring sufficient supplies of it, and the desire to avoid unnecessary consumption as also expence, other medicines were resorted to. The white and blue vitriol, and also the arsenic solution, were the medicines generally employed, and were attended with the comparative success before alluded to. Yet in many instances of the more inveterate malady, and especially in those constitutions which had suffered from the disease during the campaign in Holland, one common and general circumstance was remarked, and this was, that the disease, from the use of the above preparations, seemed to become suspended only for a time, it would sooner or later relapse, and its accession observed a period consonant to the original type of the fever, which was mostly tertian, and the relapse generally happened on the ninth day subsequent to the last paroxysm. To avoid these returns of the fever, the Peruvian bark was administered, and, after the use of either of the above preparations, was found of equal success, and effected as certain a cure as when given in other instances from the first invasion of the disease.

I certainly must coincide with Dr. Scott, whose letter in your last Journal has given rise to these observations, that the word *specific* sounds empirical, and is too generally applied; yet as the cinchona is so powerful a remedy for intermittent fever, and mercury for venereal infections, some indulgence must be allowed to those who term them by the denomination of specific. As Dr. S. does not allow those remedies which have the most claim to the title, to possess any specific quality, how can he suppose or infer, because specifics are so prevalent and fashionable, that the profession of physic is nearly at an end? It appears to me that as specifics increase, empiricism will have a proportional increment; and although quackery is daily augmenting, it seems probable that the ratio will be inversely to Dr. S's calculation, and that where there springs up one empiric there will be at least two medical officers or physicians. It gives me some satisfaction to observe, that Dr. S. prefers the cuprum ammon.* in agues, as it comes very near my own practice; large doses of ammonia I have frequently added to the medicines before noticed, and with great success.

I am, &c.

*Blackfriar's Road, Feb.* 15, 1804. C. TICE, M. D.

* The quantity of white vitriol, as prescribed by Mr. Cuming, I have frequently given to many patients in the day, yet I have found a great variety under its operation, in the constitutions of my patients. Those soldiers who had experienced the severity of the disease at the campaign in Holland, not only could not bear large doses of either of the vitriolic preparations or arsenic solution, but these medicines were mostly fallible in their operations; and in such instances, recourse to the Peruvian bark was indispensable, and which never failed of success.

